# Polyarteritis nodosa complicated by renal aneurysm and intestinal perforation: A case report

**DOI:** 10.1097/MD.0000000000039445

**Published:** 2024-08-23

**Authors:** Yingying Ma, Luan Luan, Junjun Zhang, Chunfeng Ren, Chunfeng Hou

**Affiliations:** a Department of Rheumatology, Jining No. 1 People’s Hospital, Jining, Shandong, China; b Department of Pathology, Affiliated Hospital of Jining Medical University, Jining, China.

**Keywords:** intestinal perforation, polyarteritis nodosa, renal aneurysm

## Abstract

**Rationale:**

Polyarteritis nodosa (PAN) is a necrotizing vasculitis that affects small- and medium-sized arteries, presenting with diverse clinical manifestations. It can impact tissues and organs throughout the body and may be life-threatening in severe cases. Common causes of death include cardiac, renal, and gastrointestinal complications or aneurysm rupture. While separate reports of renal aneurysm and intestinal perforation exist, the coexistence of these conditions is rarely documented. This study reports a severe case of PAN complicated by both renal aneurysm and intestinal perforation, aiming to deepen the understanding of this disease, aid in clinical diagnosis and treatment, and improve patient prognosis.

**Patient concerns:**

The patient presented to the hospital with dorsal foot pain and abdominal pain persisting for more than 4 months, along with pain and discomfort in both lower extremities for over 1 month.

**Interventions:**

The patient was diagnosed with PAN, renal aneurysm, intestinal perforation, and grade 3 hypertension (high risk).

**Outcomes:**

After treatment, the patient showed normal temperature and blood pressure, relief from abdominal pain, and disappearance of myalgia and numbness in the lower limbs. Additionally, the renal aneurysm shrank significantly, the intestinal perforation healed, the ileostomy was reduced, and the patient’s condition stabilized.

**Lessons:**

The clinical symptoms of PAN mostly lack specificity, and should be distinguished from microscopic polyangiitis and simulated vasculitis. For patients with intestinal perforation similar to this case, tocilizumab treatment may be effective, but further research is needed to confirm it.

## 1. Introduction

Polyarteritis nodosa (PAN), an inflammatory vasculitis primarily involving small- and medium-sized arteries, can cause focal segmental transmural necroinflammation, leading to arterial wall weakening and aneurysm formation. Renal involvement and gastrointestinal perforation are predictors of refractory PAN. Additionally, age over 65 years, necrotic skin lesions, and severe gastrointestinal involvement are independent predictors of recurrence.^[1,2]^ This report describes a patient with severe PAN experiencing dorsal foot pain and recurrent abdominal pain complicated by renal aneurysm and intestinal perforation. The patient’s condition improved after treatment with glucocorticoids, cyclophosphamide, and ileal perforation repair plus ileostomy.

## 2. Case report

**Figure 1.**
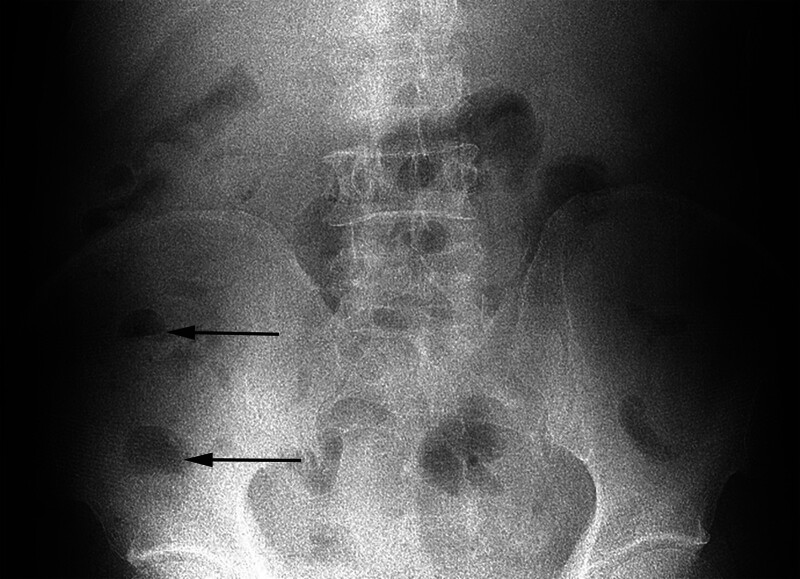
The arrow indicates gas–liquid equilibrium, suggesting intestinal obstruction.

A 58-year-old male patient was admitted to our hospital on November 12, 2018, with complaints of “left dorsal foot pain, abdominal pain for more than 4 months, and pain and discomfort in both lower limbs for more than 1 month.” Initially presenting in July 2018 with left dorsal foot pain, weakness in both lower extremities, and intermittent abdominal pain, the etiology remained unclear despite multiple medical visits. In September 2018, due to worsening abdominal pain, the patient was admitted to our hospital. Adjunctive examinations showed no abnormalities in routine blood and urine tests, liver and kidney function, with a hematocrit level of 35 mm/h, C-reactive protein (CRP) level of 34.30 mg/L, and negative antinuclear antibodies (ANA) and antineutrophil antibody (ANCA) results. An erect-position plain scan of the abdomen suggested incomplete intestinal obstruction (Fig. 1). Subsequently, the patient was admitted to the gastrointestinal surgery department and was discharged after dietary restriction, fluid replacement, and gastrointestinal decompression. Post-discharge, the patient’s abdominal pain recurred. An abdominal computed tomography (CT) performed at an outside hospital suggested small bowel dilatation and hydrops. Despite repeated visits to gastroenterology and gastrointestinal surgery departments, the abdominal pain did not significantly improve. One month ago, the patient developed pain and numbness in both lower limbs, exacerbated by walking, and occasionally numbness in both hands at night, accompanied by a low-grade fever. The diagnosis remained unclear after multidisciplinary consultation. Upon admission on November 12, 2018, further questioning revealed occasional right testicular pain, recent hypertension with systolic blood pressure reaching 180 mm Hg, and weight loss of over 20 kg in the past 4 months.

The patient was previously in good health.

Physical examination revealed a temperature of 36.6 °C, blood pressure of 180/112 mm Hg, a pain score of 5 points, and normal conscious and mental status. Cardiopulmonary auscultation did not indicate any obvious abnormalities. The abdomen was flat and soft, with light pressure pain below the umbilicus and in the left lower abdomen without rebound pain. The liver and spleen were impalpable below the costal margin, and bowel sounds were normal. The patient exhibited muscle tenderness and superficial hypoesthesia in both lower limbs, with grade 5 muscle strength in all 4 limbs.

**Figure 2.**
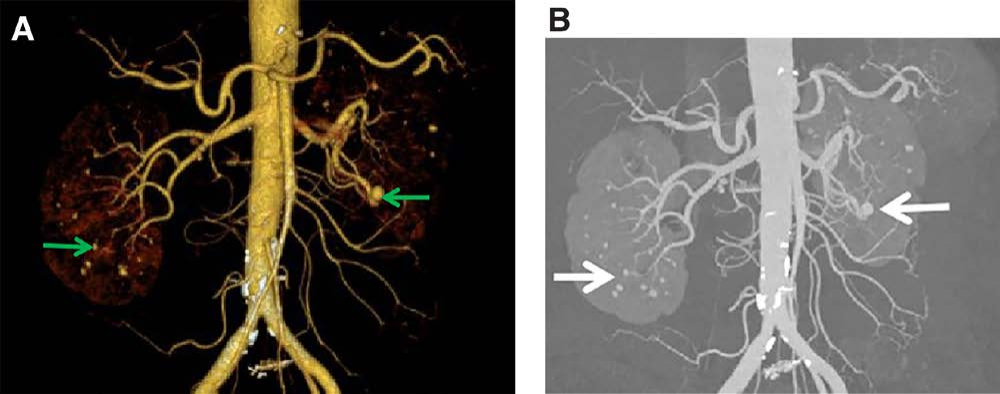
(a and b) Arrows indicate multiple distinctly enhancing nodules and possible aneurysms.

Blood routine examination indicated the following: leukocyte count of 11.80 × 10^9^/L, hemoglobin level of 106.0 g/L, platelet count of 514 × 10^9^/L, and erythrocyte sedimentation rate of 53 mm/h. Routine urine, biochemistry, and coagulation indicators were normal. Tests for hepatitis B and C antibodies were negative. The CRP level reached 111.00 mg/L. Tumor markers were negative. Stool routine examination plus occult blood test indicated positivity for occult blood. Quantitative ANA assay, ANA profile, and ANCA assay showed normal findings. Electrocardiogram revealed an incomplete right bundle branch conduction block. Electromyography indicated F-wave abnormalities in both lower limbs, suggesting suspected proximal nerve or root damage, and suspected L5/S1 segmental damage on the left side. Chest CT showed no abnormalities. Computed tomography angiography (CTA) of the upper and lower mesenteric arteries and abdominal aorta, conducted on November 14, 2018, showed calcified and non-calcified plaques on the walls of the abdominal aorta, bilateral common iliac, internal iliac, and left external iliac arteries, with mild luminal stenosis. CTA of the celiac artery and superior and inferior mesenteric arteries did not indicate significant abnormalities. Multiple markedly enhancing nodules, with some reaching up to 5 mm in diameter, were observed in both kidneys, suggesting the possibility of aneurysms. Additionally, the patient had cysts in the liver and right kidney and thickening of the gallbladder wall (Fig. 2a and b).

**Figure 3.**
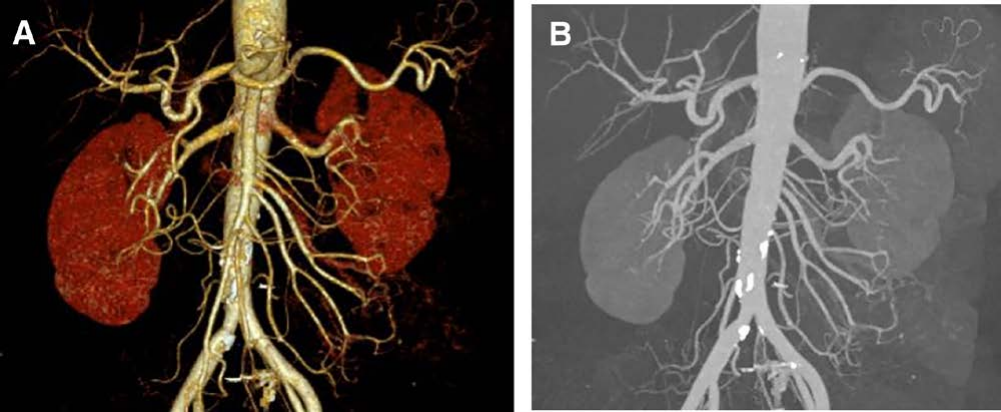
(a and b) Post-treatment CTA indicates substantial disappearance of nodules.

**Figure 4.**
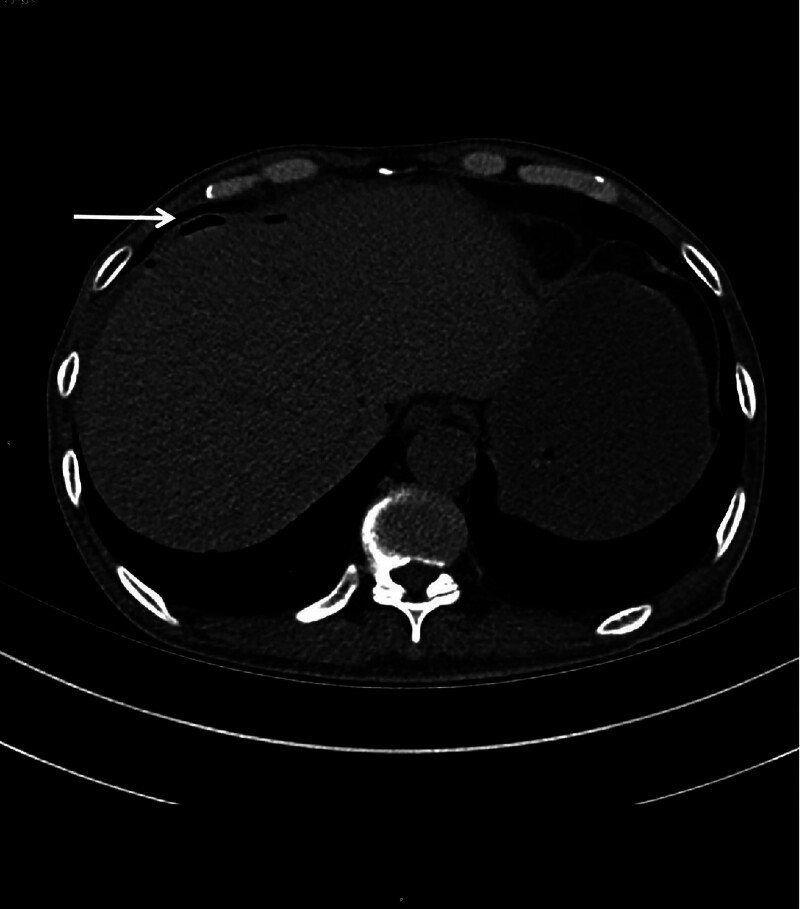
The arrow indicates subphrenic free gas, suggesting the possibility of gastrointestinal perforation.

**Figure 5.**
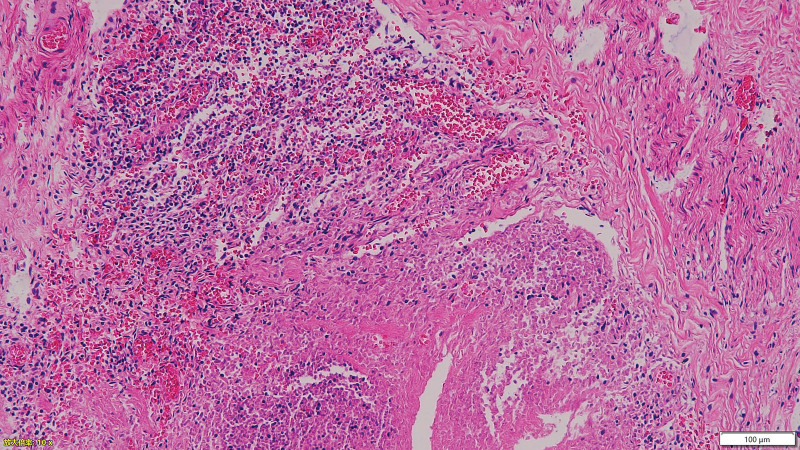
The hematoxylin–eosin staining indicates infiltration of neutrophils and monocytes at the basal level of the intestinal wall, with vasodilatation and congestion observed at 10× magnification.

The patient experienced weight loss of more than 20 kg, accompanied by testicular pain, lower limb myalgia, fatigue, nerve damage, and diastolic blood pressure over 90 mm Hg. Vascular CTA suggested a renal aneurysm. Based on these findings, the patient was diagnosed with PAN and was treated with methylprednisolone 40 mg IV daily and cyclophosphamide 0.1 g IV every other day. After the medication, the numbness in the lower limbs nearly disappeared, no fever occurred, and the blood pressure normalized. However, the abdominal pain persisted during treatment. Consequently, the methylprednisolone dose was increased to 80 mg IV daily, which improved the abdominal pain. CTA of the superior and inferior mesenteric arteries and abdominal aorta performed on December 1, 2018, revealed calcified and non-calcified plaques on the walls of the abdominal aorta, bilateral common iliac, internal iliac, and left external iliac arteries, with mild luminal stenosis. A CT scan showed no significant abnormalities in the superior and inferior mesenteric arteries but detected cysts in the liver and right kidney, thickening of the gallbladder wall, and bilateral adrenal gland thickening (Fig. 3a and b). The methylprednisolone dose was then reduced to 60 mg IV daily. On December 3, 2018, the patient experienced a paroxysmal exacerbation of abdominal pain. Physical examination revealed board-like rigidity of the abdomen, marked lower abdominal pressure, especially in the left lower abdomen, and rebound pain below the umbilicus. Blood tests showed a leukocyte count of 9.84 × 10^9^/L, erythrocyte count of 4.32 × 10^12^/L, neutrophil count of 9.17 × 10^9^/L, platelet count of 194 × 10^9^/L, amylase level of 176.00 U/L, and procalcitonin level of 0.24 ng/mL. Whole-abdomen CT indicated localized structural disorders in the small intestine, dilated small intestine in the lower left abdomen (outer diameter approximately 44 mm), and turbid and punctate gas shadows in the surrounding mesenteric fat space. Multiple fluid density shadows and free gas shadows under the diaphragm suggested signs of intestinal obstruction and possible intestinal perforation (Fig. 4). The patient was transferred to the Department of Gastrointestinal Surgery and underwent laparoscopic exploration, enterolysis, ileal perforation repair, ileal decompression, and ileostomy under general anesthesia. Intraoperatively, the small intestine was locally dilated, the intestinal wall was edematous and thickened, and the ileum had two perforations 30 cm from the proximal end. No obvious masses were observed in the abdominopelvic cavity and gastrointestinal tract. The postoperative diagnosis was ileal perforation with acute diffuse peritonitis. Pathology suggested neutrophilic and mononuclear cell infiltration at the base of the intestinal wall, with vasodilatation and congestion (Fig. 5). Postoperatively, glucocorticoids were suspended, and the patient received human intravenous immunoglobulin 20 g IV daily for 3 days and cyclophosphamide 0.2 g IV every other day, supplemented with anti-infection and nutritional support. The patient’s condition improved, and on the 10th postoperative day, prednisone 20 mg daily was added orally. The patient’s abdominal pain resolved, no fever occurred, the incision healed well, and there was no discomfort after eating. The patient was discharged on December 25, 2018. After discharge, the patient continued to take cyclophosphamide 100 mg every other day and a tapering dose of prednisone. Multiple erythrocyte sedimentation rate, CRP, liver and kidney function, and routine blood tests showed normal conditions. The patient stopped taking prednisone on July 15, 2019. In August 2019, the patient underwent ileostomy reduction in our gastrointestinal surgery department. He recovered well after the operation and underwent regular reexaminations. As of April 15, 2024, the patient has been followed up routinely, taking cyclophosphamide tablets 50 mg every other day, and has not experienced any further abdominal or lower extremity pain. No abnormalities in inflammatory markers have been detected.

## 3. Discussion

The patient initially presented with recurrent abdominal pain and lower extremity pain. During the course of the disease, hypertension, significant weight loss, and testicular pain developed, accompanied by renal aneurysm, nerve damage in the lower extremities, and intestinal perforation, indicating multisystem involvement. Intestinal pathology revealed neutrophilic and mononuclear cell infiltration. The patient met 7 of the 10 classification criteria of the 1990 American College of Rheumatology for Polyarteritis Nodosa, leading to a diagnosis of PAN. However, some patients classified as PAN according to the 1990 ACR classification criteria may be reclassified as microscopic polyangiitis (MPA) under the new classification system, therefore requiring further differential diagnosis. According to the 2022 ACR/EULAR MPA classification criteria,^[3]^ the patient had no lung and renal involvement, and ANCA was negative, which excluded the diagnosis of MPA. In addition, deficiency of adenosine deaminase 2 (DADA2) can present with vasculitis manifestations similar to PAN, which should be carefully differentiated. DADA2 is an autosomal recessive disease caused by the loss-of-function mutations in the cat eye syndrome chromosome candidate gene 1. DADA2 is seen in both children and adults, but most DADA2 cases occur in childhood. Adult-onset cases have occasionally been reported. The clinical features of this disease include vasculitis/vasculopathy, immunodeficiency, autoimmunity, autoinflammation, and hematological manifestations. Initially, it was described as vasculopathy/vasculitis and closely resembled PAN.^[4,5]^ Given its similarity to PAN, DADA2 should be carefully differentiated, particularly in cases of early-onset PAN, familial PAN, PAN with stroke, and treatment-refractory or poorly-responsive PAN.^[6]^ Since the disease is predominantly observed in children, and considering that the patient is an elderly male with no symptoms such as stroke, blood system involvement, or immunodeficiency, whose condition remains stable during long-term follow-up, DADA2-related testing was not performed.

Up to 75% of patients with PAN may experience kidney involvement, characterized by arterial stenosis and aneurysms. Typical presentations include hematuria, proteinuria, recent hypertension, and renal infarction. Angiography in patients with renal involvement may show renal infarction, multiple stenoses, and/or microaneurysms. The incidence of aneurysms in patients with PAN increases with clinical severity. Serious complications such as aneurysm rupture and spontaneous perirenal hemorrhage may be life-threatening and require embolization or nephrectomy.^[7–9]^ Due to the potential risk of rupture and hemorrhage of microaneurysms, ultrasound-guided renal/hepatic biopsy is not the diagnostic choice unless the diagnosis cannot be confirmed by other means. The diagnosis of PAN is supported when CT or angiography shows characteristic changes, such as saccular or spindle-shaped microaneurysms (1–5 mm in diameter) involving the bifurcations and branches of small- and medium-sized arteries.^[10,11]^ The evolution of multiple vascular aneurysms is the most typical angiographic manifestation of PAN; follow-up imaging is particularly important when baseline imaging shows aneurysms.^[12,13]^ The initial vascular CTA in our patient suggested a renal aneurysm. After treatment with glucocorticoids and cyclophosphamide, the renal aneurysm was significantly reduced, supporting the diagnosis of PAN complicated by renal aneurysm.

Gastrointestinal manifestations of PAN are common, affecting up to 50% of patients, and are associated with a high mortality rate. Mild cases may present with abdominal pain, typically manifesting as postprandial pain or abdominal cramps. Severe vascular inflammation of the mesenteric arteries can lead to intestinal ischemia, perforation, and bleeding. In severe cases, ischemic enteritis presents with hematochezia, and transmural intestinal wall ischemia can lead to perforation. Additionally, 10% of patients with PAN have experienced gastrointestinal bleeding or required abdominal surgery due to gastrointestinal complications.^[9,14,15]^ Most patients experience small bowel ischemia, while colon or stomach ischemia is rare, with small bowel perforation and gastrointestinal bleeding being the most severe clinical manifestations.^[7]^ This patient’s initial symptom was abdominal pain, accompanied by recurrent intestinal obstruction and abdominal pain. The abdominal pain improved after glucocorticoid treatment. However, small bowel perforation occurred during hormone tapering over more than 20 days of treatment. Pathologic examination of the intestinal wall indicated acute and chronic inflammatory cell infiltration. The patient healed well after treatment with glucocorticoids, cyclophosphamide, and gammaglobulin, suggesting the perforation was likely due to ischemic enteritis in PAN. Additionally, patients with PAN may present with gallbladder involvement, malabsorption with weight loss, and pancreatitis. In rare but severe cases, hepatic aneurysms can occur, potentially triggering acute liver failure and leading to death.^[7,9]^

Glucocorticoids are regarded as the first-line therapeutic agents for PAN, providing rapid relief of neurologic and systemic symptoms, controlling vascular inflammation, and reducing complications. For patients with newly diagnosed active severe PAN, it is conditionally recommended to initiate treatment with cyclophosphamide and high-dose glucocorticoids rather than high-dose glucocorticoids alone. Immunosuppressive agents such as azathioprine, methotrexate, chlorambucil, cyclosporine, mycophenolate mofetil, and leflunomide may also be used in addition to cyclophosphamide.^[16,17]^

Regarding refractory PAN, it has been reported that the application of interleukin-6 (IL-6) receptor inhibitor tocilizumab can effectively control disease activity. It may be related to various pro-inflammatory processes mediated by IL-6 and its involvement in the pathogenesis of systemic inflammatory diseases. Early studies have shown that the level of IL-6 in blood (cutaneous) PAN is correlated with disease activity, making the IL-6 signaling pathway a potential target in the clinical management of PNA.^[18,19]^ In addition, there were cases reported that patients who did not respond to IL-6 treatment received good efficacy with tofacitinib.^[20]^ Tumor necrosis factor inhibitors are reported to be effective in pediatric PAN and adult (cutaneous) PAN patients.^[21,22]^

## 4. Conclusion

In conclusion, PAN is difficult to diagnose due to its diverse clinical manifestations and often lack of specificity owing to the involvement of different arterial locations. For PAN patients with early-onset, familial history or poor treatment response, testing for the cat eye syndrome chromosome candidate gene 1 and assessing ADA2 enzyme activity can assist in the diagnosis of DADA2 and facilitate timely treatment with tumor necrosis factor inhibitors. For patients with intestinal perforation similar to this case, if the cause of perforation is not confirmed through intestinal pathological examination, the possibility of adverse reactions to glucocorticoids cannot be ruled out, thereby limiting the use of glucocorticoids. According to the current study, we speculate that treatment with tocilizumab may be effective; however, further research is needed to confirm its efficacy.

## Author contributions

**Conceptualization:** Yingying Ma, Chunfeng Hou.

**Data curation:** Yingying Ma, Luan Luan, Junjun Zhang.

**Project administration:** Chunfeng Hou.

**Writing – original draft:** Yingying Ma.

**Writing – review & editing:** Chunfeng Ren, Chunfeng Hou.
